# Dental Caries

**Published:** 1880-06

**Authors:** Louis R. Ebert

**Affiliations:** Rio de Janeiro.


### ARTICLE V.

### Dental Caries.

BY DR. LOUIS R. EBERT, OF RIO DE JANERIO.

The destruction of tooth substance is attributed to acids and leptothrix by Magitot, Leber and Rottenstein, etc. There are authors who also attribute dental caries to an animated cause, producing to teeth and organs necessary to health, sad ravage and pain. Now, the true cause of dental caries not having been as yet thoroughly elucidated, becomes to all interested a subject of minute observation and close study. Having lately devoted much time to serious research in this direction, we find ourself more and more confirmed in the belief that there exists *in certain cases of caries* an animated agent, microscopic in size, and having mandibles.

There is a special species attacking the necks of the incisors, eye teeth, bicuspids, and sometimes, but very seldom, in the lower jaw. This latter decay is certainly caused by an acid coming from either the secretions of the mucous membrane or the parotid glands, having no cororation, and the caried part bears exact resemblance to that placed in a tooth in acids; the march of the decay proceeding internally as well as superficially, having a regular border and entirely free tooth particles.

The point of attack for caries, to whose cause we attribute an animated organ, is first perceptible by a small dark point in the slight hollow on the labial surface of the molars in the interstices of their grinding surface as well as those of the bicuspids, in the marked groove of the upper incisors on the palatine surface; between the teeth, where food remains lodged, and which, in the stage of fermentation and decomposition does not produce enough of acid, but certainly enough of vibrions, etc., to produce damage, while seldom if ever will decay begin on the lingual surface of the lower maxillary, while sometimes the second molar of the upper jaw will have the point of commencement on the palatine surface, which proves clearly that acids are not exclusively the cause. The liquids of the mouth cannot find a spot of attachment, because kept in constant circulation by the natural movement of the tongue, muscles and cheeks..

It remains yet for the microscope of superior power to the one employed by Leber and Rottenstein to prove that his leptothrix are but adjuncts instead of causes of dental caries.

The opinion expressed by Raspail on dental caries is far from being exaggerated in that which concerns their cause, formation and aspect, and comes in to justify our preceding assertions in regard to caries of the molars and bicuspids, showing clearly to the observer its point of attack where the tongue cannot reach it and where it cannot be crushed by the antagonizing teeth in the labor of mastication; then quietly and safely proceeding in its work of destruction, fully protected, it perforates internally in the direction of the nerve, hollowing out the tooth, leaving untouched the walls of enamel that by accident and mastication crumble down, displaying to the surprise of the owner an immense cavity that had been forming without attracting his attention in the slightest degree. Daily do patients call on us expressing surprise at a tooth having been crushed in or at one which all of a sudden gives them intense pain.

As regards the small worms, of which Scribnus Largus treats, and which seemed to have been seen by Bremer and Brera in the saliva expectorated by persons suffering from odontalagia and in those to whom they gave narcotic plants to inhale, they are probably the same of which Ficinus speaks, who attributes dental caries to a putrefaction produced by small infusori living in the mouth and found in large numbers in the tartar that covers the teeth. It is more probable, we think, that these infusoria and vibrons have nothing to do with caries, but help to form the salivary calculus that, according to Mandl, is composed of calculary carapaces.

According to observations that we have made, the destructive agent makes its way internally at the points of which we have already spoken. Nature furnishes us analogous instances of work by a certain class of mollusks that perforate the hardest of rocks, either by a corrosive or perforating power. As to the disappearance of the calcarious salts of which some authors speak, we again find like agents in nature, not only plants but sea animals, which make it their nutritive element. It may be that this agent, when once in the pulp chamber, extracts voraciously the juices needful for his sustenance, and causes odontalagia, which those suffering from it often describe as something gnawing and biting the nerve.

Before the work of destruction has gone far, the cavity is found full of small microscopic particles, which clearly show that they have been detached, and fall on the tongue or napkin in a perfectly dry state. This would be otherwise if they were produced by acids. These particles are not produced by the excavator nor bur, and certainly no work of disorganization can produce them.

There arrives a stage in the disease, however, as we have already observed, when the walls of the tooth, partially or entirely hollowed out, break in, giving full ingress to the fluids of the mouth, and then the acids play an active part, aiding frightfully in the work of destruction and changing the aspect of the cavity.

Van Beneden tells us clearly in his work, "Commensaux et Parasites," that neither the brain, ear, eye, heart, blood, lungs, nor the spinal marrow, nor the nerves, nor the muscles, not even the bones are exempt from attacks of parasites.

Now, could the teeth be the only organ in the human frame excepted from the attack of parasites? Certainly not; for, by their mere form, position and contact with parasite forming agents they are more subject than any other organs. At different times examinations have been made, by men in the profession, on caries produced on teeth placed in jars in which were liquids and substances that produced caries similar to those found in the mouth. We cannot accept these results as conclusive, for a tooth thus exposed is not in its normal state and cannot offer the same points of observation ; nor can it be compared to a tooth placed with its companions in the oral cavity, constantly subjected to sudden thermal changes, constantly in contact with liquids more or less charged with acid or animal life, having a living nerve-giving life, by means of the analculi, and which may, by the mere supply of poor or vitiated blood, transmit but feeble elements for its strength, thus offering less resistance to the attacking agent. Persons having soft teeth have them far more decayed than persons of sound, hard teeth, having the same elements and liquids for food and drink.

The observations on dental caries must be made *seance tenante* or immediately after extraction, which operation, fortunately for the patient, is only resorted to when the tooth is all destroyed or too far gone for the minute research so necessary in the first stage of its destruction.

We are often very much surprised at the rapidity of decay in some teeth while other teeth in the same mouth remain intact. Why is this? Are they not continually bathed by the same secretions, subject to the same thermal changes? And if the cause of decay be acid, not one but all the teeth would be more or less attacked.

Science having clearly proved that nearly all our organs are subject to the attacks of parasites, we are more and more convinced that the teeth are not exceptions, especially as we have, in our late researches, found what appears to be the destructive agent lodged in the cavities, of which we have made mention.

But one cannot continue this subject, on which so much has been said and so much labor expended, without the presentation of unrefutable results, without more perfect instruments.—*Dental Miscellany.*

